# Correlation between lung infection severity and clinical laboratory indicators in patients with COVID-19: a cross-sectional study based on machine learning

**DOI:** 10.1186/s12879-021-05839-9

**Published:** 2021-02-18

**Authors:** Xingrui Wang, Qinglin Che, Xiaoxiao Ji, Xinyi Meng, Lang Zhang, Rongrong Jia, Hairong Lyu, Weixian Bai, Lingjie Tan, Yanjun Gao

**Affiliations:** 1 Department of Radiology, Xi’an No.3 Hospital, The Affiliated Hospital of Northwest University, Shaanxi Province 710018 Xi’an, China; 2 Xi’an Key Laboratory of Cardiovascular and Cerebrovascular Diseases, Xi’an No.3 Hospital, the Affiliated Hospital of Northwest University, Northwest University, Xi’an, 710018 Shaanxi Province China; 3 Department of Radiology, Jingmen No.1 People’s Hospital, Jingmen, 448000 Hubei Province China

**Keywords:** COVID-19, SARS-CoV-2, Artificial intelligence, Deep learning, Lymphocyte, Neutrophil, Infection severity

## Abstract

**Background:**

Coronavirus disease 2019 (COVID-19) has caused a global pandemic that has raised worldwide concern. This study aims to investigate the correlation between the extent of lung infection and relevant clinical laboratory testing indicators in COVID-19 and to analyse its underlying mechanism.

**Methods:**

Chest high-resolution computer tomography (CT) images and laboratory examination data of 31 patients with COVID-19 were extracted, and the lesion areas in CT images were quantitatively segmented and calculated using a deep learning (DL) system. A cross-sectional study method was carried out to explore the differences among the proportions of lung lobe infection and to correlate the percentage of infection (POI) of the whole lung in all patients with clinical laboratory examination values.

**Results:**

No significant difference in the proportion of infection was noted among various lung lobes (*P* > 0.05). The POI of total lung was negatively correlated with the peripheral blood lymphocyte percentage (L%) (*r* = − 0.633, *P* < 0.001) and lymphocyte (LY) count (*r* = − 0.555, *P* = 0.001) but positively correlated with the neutrophil percentage (N%) (*r* = 0.565, *P* = 0.001). Otherwise, the POI was not significantly correlated with the peripheral blood white blood cell (WBC) count, monocyte percentage (M%) or haemoglobin (HGB) content. In some patients, as the infection progressed, the L% and LY count decreased progressively accompanied by a continuous increase in the N%.

**Conclusions:**

Lung lesions in COVID-19 patients are significantly correlated with the peripheral blood lymphocyte and neutrophil levels, both of which could serve as prognostic indicators that provide warning implications, and contribute to clinical interventions in patients.

**Supplementary Information:**

The online version contains supplementary material available at 10.1186/s12879-021-05839-9.

## Background

In December 2019, several cases of pneumonia of unknown causes were reported in Wuhan, Hubei Province, China [1]. It was confirmed that the pneumonia was coronavirus disease 2019 (COVID-19), which was caused by a new coronavirus. Later, the International Committee on Taxonomy of Viruses (ICTV) officially named the virus severe acute respiratory syndrome coronavirus type 2 (SARS-CoV-2). Studies have shown that SARS-CoV-2, which is a beta genera coronavirus, shares similarity with the viruses that cause SRAS and Middle East respiratory syndrome (MERS); however, SARS-CoV-2 is more severely contagious [2]. SARS-CoV-2 is principally transmitted through respiratory droplets (cough, sneeze) and contact, and people of all ages are generally susceptible. The symptoms after infection mainly include fever, dry cough, and fatigue. Most patients have a comparatively good prognosis, and a few quickly progress to acute respiratory distress syndrome (ARDS), sepsis shock, and multiple organ failure, which heralds a poor prognosis. On March 11, 2020, the WHO announced that COVID-19 has caused a global pandemic. As of March 15, 2020, COVID-19 had spread in 135 countries and territories around the world, with more than 330,000 cumulative confirmed cases and more than 14,000 deaths [3].

COVID-19 generally attacks within 14 days after infection, and its diagnosis depends on viral nucleic acid testing, which is susceptible to interference by some factors. Moreover, the sensitivity of nucleic acid testing is relatively lower than that of computer tomography (CT) (71% vs. 98%) [4]. CT is recommended for clinical screening and observation of COVID-19 patients due to its high efficiency and objectivity. However, visual inspection of CT imaging cannot attain a quantitative assessment of the infected area and is incapable of accurately judging the patient’s progress. At present, artificial intelligence (AI) technology is becoming increasingly mature. This technology is adept in automatically identifying complex patterns in imaging data, can quantitatively evaluate specific imaging features, and has been widely used in the field of medical imaging [5].

COVID-19 mortality is lower but its morbidity is higher compared with SARS and MERS [6, 7]. Current evidence shows that the primary diseased region of COVID-19 patients is the lungs, and normal or decreased peripheral leukocytes, as well as reduced lymphocyte counts, are noted [8, 9]. Based on the current diagnostic sensitivity of high-resolution CT (HRCT), early changes in the lungs of COVID-19 patients are easily detected. To ensure the accuracy of diagnosis, it is necessary to combine CT results with clinical laboratory testing indicators. However, there is a lack of evidence that indicates the exact relationship between COVID-19 progression and laboratory testing indicators. To further clarify the dynamic changes of relevant clinical laboratory test indicators and their significance in the diagnosis and treatment of COVID-19, this study intends to use AI technology to quantitatively evaluate the extent of pulmonary lesions in COVID-19 patients and, in combination with their respective blood observation indexes, to explore the correlation between the two factors, so as to provide a clinical reference.

## Methods

### Study design

Chest HRCT images and laboratory examination data of COVID-19 patients were extracted, and lesion areas in the CT images were quantitatively segmented and calculated using the deep learning (DL) system. A cross-sectional study was conducted to investigate the correlation between lung infection and clinical laboratory indicators in patients with COVID-19 pneumonia.

### Participants

Thirty-one patients with a diagnosis of COVID-19 were collected from January 21, 2020, to February 4, 2020, in Jingmen No.1 People’s Hospital, Hubei Province, China. All patients received a respiratory or blood specimen test via fluorescent reverse-transcription polymerase chain reaction (RT-PCR), and the results were positive for new coronavirus nucleic acids. Furthermore, the viral gene sequence must be highly homologous to the new coronavirus sequence. All participants underwent a HRCT scan and peripheral blood laboratory testing on the same day without other basal diseases that may affect laboratory observation indicators (such as combined bacterial infection and immunosuppression).

In order to comprehensively evaluate the severity of the included patients, the clinical classification of each patient was determined based on his (her) clinical manifestations, radiological and laboratory examination results to serve as a supporting data for the degree of pulmonary infection. The new coronavirus pneumonia diagnosis and treatment plan (trial version 7) (ncpDTP-7) developed by the National Health Commission of the People’s Republic of China [10] was used as a diagnostic standard. These patients have not been reported in any other submission by anyone else.

### Imaging data acquisition and post-processing

HRCT images were collected in the Department of Radiology, Jingmen No.1 People’s Hospital, Hubei Province. A GE MEDICAL SYSTEMS LightSpeed ​​VCT scanner was used. All patients were supine, and the images were captured after the patients were instructed to hold his (her) breath. The following scanning parameters were employed: slice thickness, 1.25 mm; field of view (FOV), 354.0 mm; tube voltage, 120 kV; tube current, 278 mA; and image zoom, 1.00. The AI ​​analysis software used for image processing was a deep learning system developed by Shanghai United Imaging Intelligence Co., Ltd. and Shanghai Public Health Clinical Center, Fudan University (New Coronavirus Pneumonia Auxiliary Analysis Software, version number: Full-uAI-Discover-NCP.R001.0.0.15980) [11]. The lung window (with window width 1200 HU and window level − 600 HU) was used for image reading.

### Laboratory inspection data collection

We scrutinized the clinical data of all laboratory-confirmed COVID-19 patients in the in-hospital medical records system, including clinical charts, laboratory test results, radiological diagnosis opinions and nursing records, and extracted the clinical laboratory examination indicators of each patient through standardized data collection. After the collection of clinical laboratory inspection indicators, the data were independently reviewed and checked by two researchers to ensure that the relevant values were accurate.

### Observation indicators

The original HRCT images of 31 patients were extracted from the Picture Archiving and Communication System (PACS), and all patients were randomly numbered for identification (patient 1, patient 2, ..., patient 31). The lungs of each patient were divided into 20 bronchopulmonary segments based on anatomical division by DL system, and lesions of the whole lung and each lung lobe were calculated. The infection regions were determined through identifying ground-glass opacity (GGO) (− 750 HU to − 300 HU) and consolidation component (− 300 HU to 50 HU) in the lungs. Besides, a small number of voxels with Hounsfield unit (HU) values not falling within the interval [− 750, 50] were surrounded by GGOs or consolidation area, which were also designated as the infected area by the system [11]. Specific steps: 1) input the HRCT images into the DL automatic segmentation system; 2) calculate the quantitative metrics that characterize infected regions, including but not limited to the volume of infection (VOI) and the percentage of infection (POI) in the whole lung, lung lobes and bronchopulmonary segments.

**Fig. 1:**
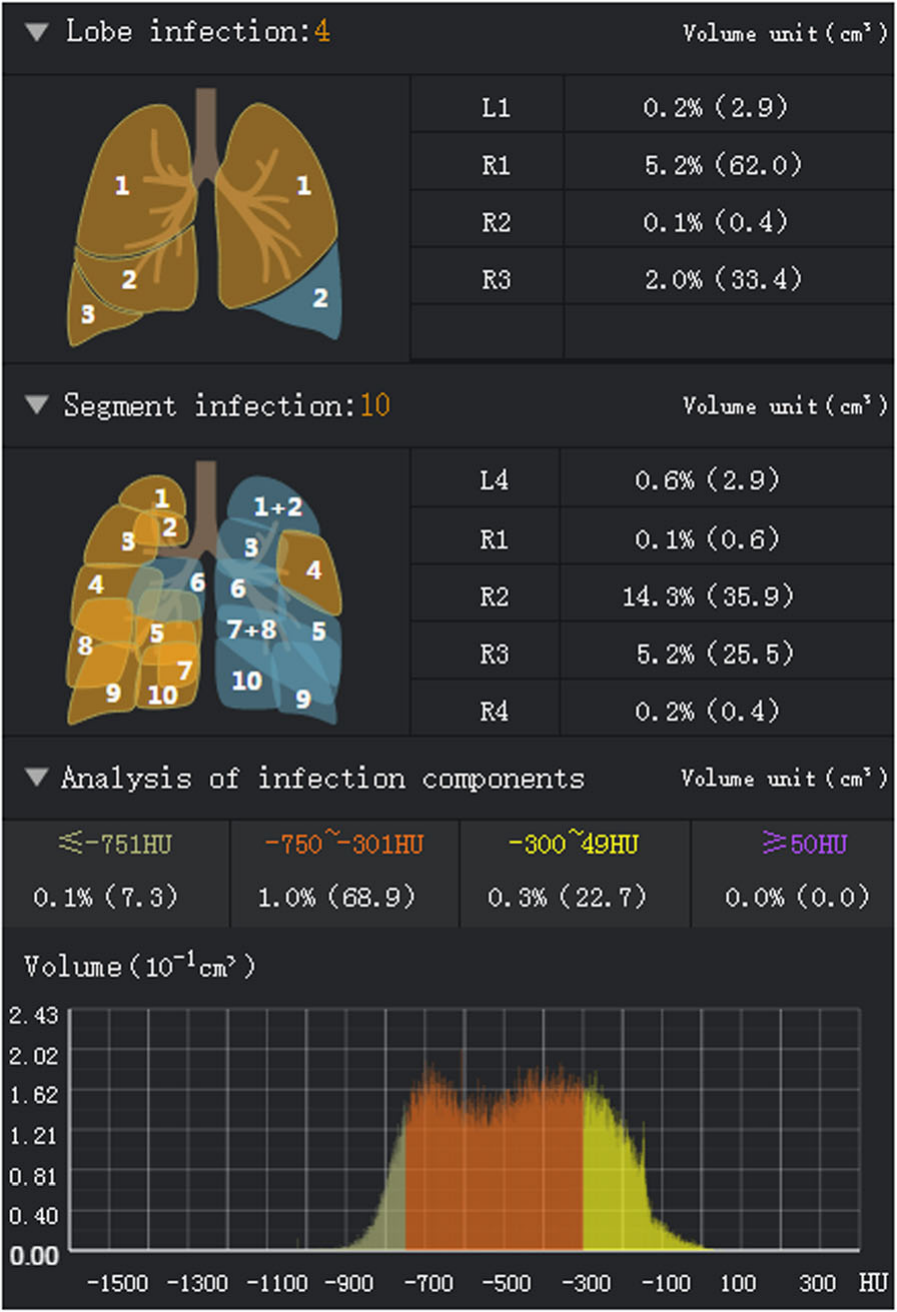
Software interface obtained by inputting original HRCT images of one patient into the DL system. The VOIs and POIs in the lung lobes and bronchopulmonary segments are presented; the HU histogram within the infection regions can be visualized

**Fig. 2:**
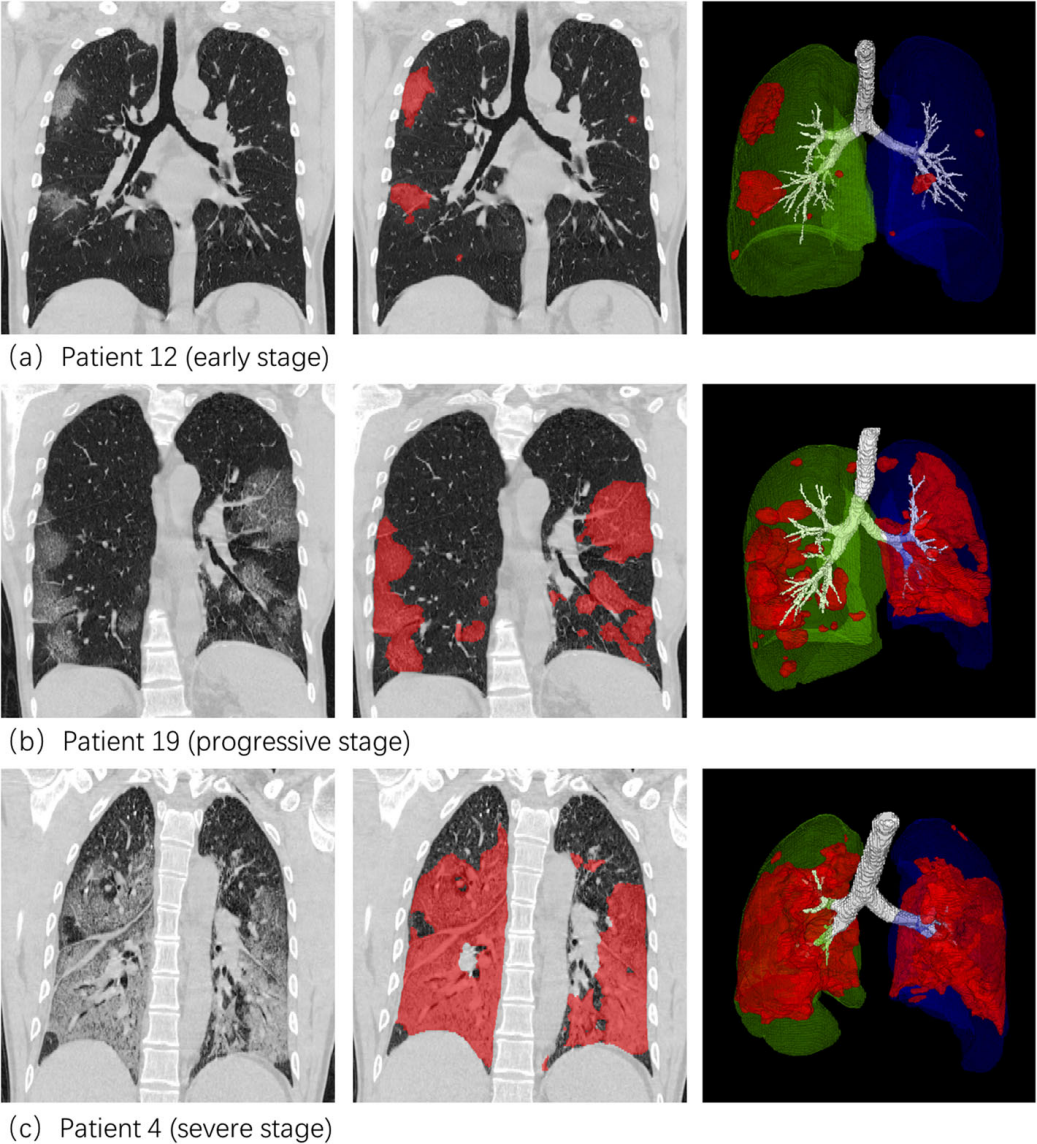
Lesion segmentation results of three COVID-19 cases displayed using the software post-processing platform. Rows 1–3: early, progressive, and severe stages. Columns 1–3: CT image, CT images overlaid with segmentation, and 3D surface rendering of segmented infections

Figure 1 shows the software interface obtained by inputting the original HRCT images of one patient into the DL automatic segmentation system. The HU histogram within the infection regions can be visualized. Figure 2 shows the CT image segmentation results of typical COVID-19 infection cases at three different infection stages: the early stage, progressive stage, and severe stage. The contour drawn by the DL system coincided with the lesion boundary visible in the CT image.

The peripheral blood white blood cell (WBC) count, neutrophil percentage (N%), lymphocyte percentage (L%), monocyte percentage (M%), lymphocyte (LY) count, and haemoglobin (HGB) content were extracted. Laboratory examination and HRCT image acquisition of each patient were completed on the same day. Two independent researchers verified all the values.

### Statistical analysis

The Shapiro-Wilk test was used to verify the normality of the data. The Kruskal-Wallis test was performed on the POIs of the following five lung lobes: the left upper lobe, left lower lobe, right upper lobe, right middle lobe, and right lower lobe. The Spearman correlation test was carried out to analyse the correlation between the total pulmonary POI and the peripheral blood WBC count, N%, L%, M%, LY count, and HGB content. SPSS 19.0 statistical software (IBM Company, Armonk, NY) was employed.

## Results

A total of 31 COVID-19 patients were involved in this study, including 18 males (58.1%) and 13 females (41.9%). The patients were 17 to 80 years old with an average age of 42.6 ± 16.0 years. Of the 31 patients, 8 (25.8%) had one or more comorbidities. The most common symptoms at the initial stage of illness were fever [29 (93.5%)], cough [15 (48.4%)]. Less common symptoms were fatigue [2 (6.5%)], myalgia [1 (3.2%)] and diarrhea [1 (3.2%)]. The average interval from illness onset to CT scan was 8.8 ± 5.7 days, and the mean duration of hospitalization before the CT scan was 2.6 ± 3.5 days. The average proportion of affected lungs calculated by AI was 8.91% ± 15.01%. Thereinto, 21 patients (67.7%) had an infection of less than 5%; 5 patients (16.1%) were between 5 and 20%, the other 5 (16.1%) were for more than 20%. According to the criteria in ncpDTP-7, 23 patients (74.2%) were moderate, 4 (12.9%) were severe and remaining 4 (12.9%) met the critical type. More demographic data and laboratory tests of the study group are listed in Table 1.

**Table 1:** Demographics, clinical characteristics, and laboratory findings of COVID-19 patients

Characteristics	
Age, years	42.6 ± 16.0
< 40	16 (51.6%)
40–60	12 (38.7%)
> 60	3 (9.7%)
Sex	
Female	13 (41.9%)
Male	18 (58.1%)
Hypertension/ diabetes /cardiovascular disease/ cerebrovascular disease/COPD/kidney disease	8 (25.8%)
Signs and symptoms	
Fever	29 (93.5%)
Cough	15 (48.4%)
Fatigue	2 (6.5%)
Myalgia	1 (3.2%)
Diarrhea	1 (3.2%)
Interval from illness onset to CT scan, days	8.8 ± 5.7
Days of clinical intervention before CT scan	2.6 ± 3.5
Proportion of affected lungs calculated by AI (%)	8.91 ± 15.01
< 5%	21 (67.7%)
5–20%	5 (16.1%)
> 20%	5 (16.1%)
Clinical classification according to ncpDTP-7	
Mild	0 (0.0%)
Moderate	23 (74.2%)
Severe	4 (12.9%)
Critical	4 (12.9%)
Arterial blood gas on the day of CT scan	
PaO_2_, mmHg	57.7 ± 19.8
< 60	4/6 (66.7%)
≥ 60	2/6 (33.3%)
PaCO_2_, mmHg	42.7 ± 6.8
≤ 50	5/6 (83.3%)
> 50	1/6 (16.7%)
Laboratory findings	
White blood cell count, × 10^9^/L	4.80 ± 1.87
< 4	13 (41.9%)
4–10	17 (54.8%)
> 10	1 (3.2%)
Lymphocyte percentage (%)	30.3 ± 13.3
Lymphocyte count, ×10^9^/L	1.35 ± 0.59
Neutrophil percentage (%)	57.4 ± 14.1
Monocyte percentage (%)	11.0 ± 3.7
Hemoglobin, g/L	131.4 ± 18.1
AST, U/L	35.3 ± 28.6
≤ 40	21/30 (70.0%)
> 40	9 /30 (30.0%)
ALT, U/L	26.7 ± 18.4
≤ 35	25/30 (83.3%)
> 35	5 /30 (16.7%)
CRP, mg/L	19.2 ± 30.1
≤ 5	10/21 (47.6%)
> 5	11/21 (52.4%)
CK, U/L	148.3 ± 271.9
≤ 200	25/28 (89.3%)
> 200	3/28 (10.7%)

Continues data are expressed as mean ± SD. Categorical data are presented as n (%) or n/N (%), where N is the total number of patients with available data

Abbreviations: COVID-19 coronavirus disease 2019, COPD chronic obstructive pulmonary disease, CT computer tomography, AI artificial intelligence, ncpDTP-7 new coronavirus pneumonia diagnosis and treatment plan (trial version 7), AST aspartate transaminase, ALT alanine transaminase, CRP C-reactive protein, CK creatine kinase

**Fig. 3:**
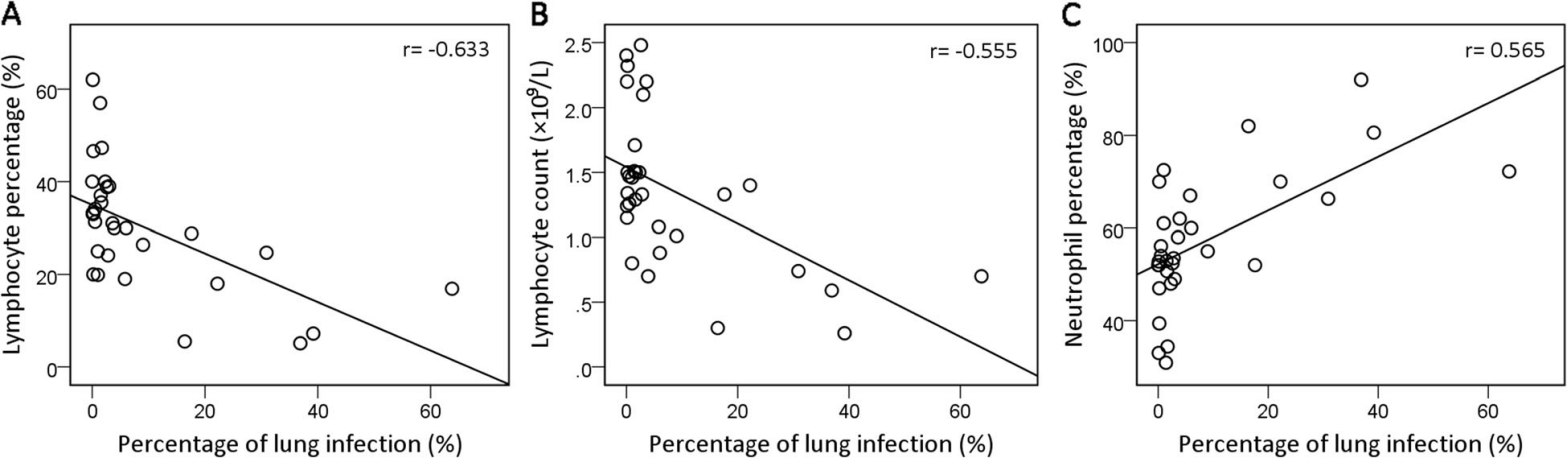
Correlation between total pulmonary POI and L%, LY count, or N%. POI of the total lung was negatively correlated with the L% [*r* = −0.633, *P* < 0.001, (**a**)] and LY count [*r* = − 0.555, *P* = 0.001, (**b**)] but positively correlated with the N% [*r* = 0.565, *P* = 0.001, (**c**)]

The status of pulmonary infection is presented in Table 2. No significant difference was noted among the proportions of pulmonary lobe infection (*P* > 0.05). Correlation analysis found that the total pulmonary POI was negatively correlated with the L% (*r* = − 0.633, *P* < 0.001, Fig. 3a) and the LY count (*r* = − 0.555, *P* = 0.001, Fig. 3b) but positively correlated with the N% (*r* = 0.565, *P* = 0.001, Fig. 3c). The peripheral blood WBC count, M% and HGB content did not significantly correlate with the total POI of the lungs (Table 3).

**Table 2:** Lung (lobes) average infection volume and proportion

Anatomical partition	VOI (cm^3^)	POI (%)
Whole lung	281.19 ± 421.55	8.91 ± 15.01
Left lung	115.45 ± 191.99	7.81 ± 13.26
Upper lobe	57.02 ± 108.02	7.72 ± 14.92
Lower lobe	58.44 ± 93.80	10.31 ± 20.26
Right lung	165.73 ± 243.46	9.98 ± 17.14
Upper lobe	54.39 ± 89.82	9.50 ± 18.50
Middle lobe	20.44 ± 39.93	8.20 ± 18.54
Lower lobe	90.91 ± 123.99	13.54 ± 22.82

Data are expressed as mean ± SD

Abbreviations: VOI volume of infection, POI percentage of infection

**Table 3:** Correlation between total lung infection and the clinical laboratory indicators

	POI of whole lung	
	Correlation coefficient (*r*)	*P-*value*
Lymphocyte percentage	−0.633	0.000
Lymphocyte count	−0.555	0.001
Neutrophil percentage	0.565	0.001
White blood cell count	0.057	> 0.05
Monocyte percentage	0.097	> 0.05
Haemoglobin content	−0.193	> 0.05

*P < 0.05 was considered statistically significant

Abbreviations: POI percentage of infection

**Fig. 4:**
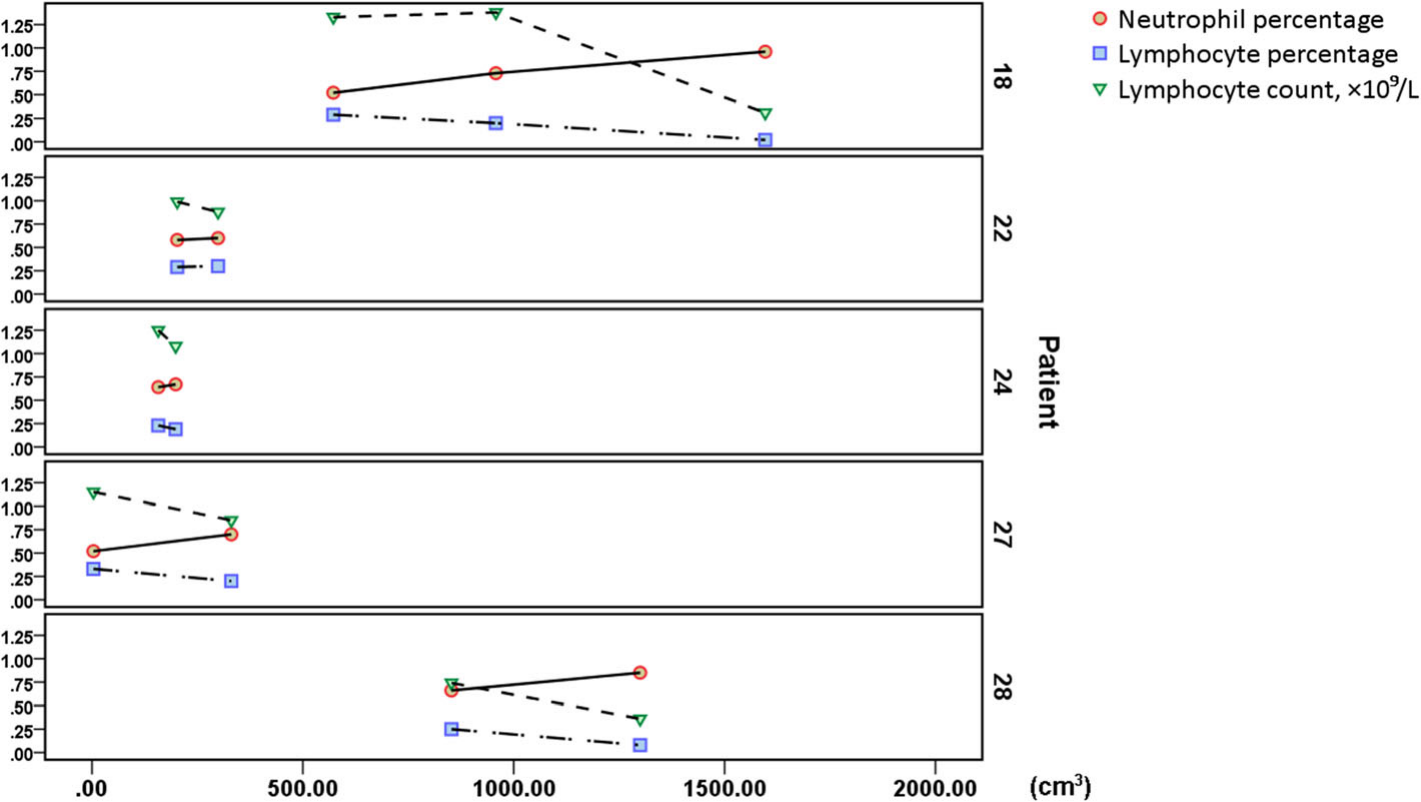
Dynamic trend of L%, LY count, and N% in several patients. As the pulmonary infection volume increased, L% and LY count exhibit progressively decreased accompanied by continuously increased N%

In the data we collected, of note, several patients in different disease periods showed a dynamic trend of progressively decreased L% and LY count, accompanied by continuously increased N%, which correlated with the increase in the pulmonary infection volume (Table 4, Fig. 4).

**Table 4:** Dynamic changes of the lung infection volume and laboratory indicators in some patients

	Infected lung volume (cm^3^)	Neutrophil percentage (%)	Lymphocyte percentage (%)	Lymphocyte count (×10^9^/L)
Patient 18				
Time 1	572.2	52.0	28.8	1.33
Time 2	957.7	72.5	19.5	1.38
Time 3	1596.4	95.9	1.7	0.31
Patient 22				
Time 1	202.7	57.8	28.6	0.99
Time 2	298.8	60.2	30.3	0.88
Patient 24				
Time 1	157.1	64.0	23.4	1.25
Time 2	198.3	67.2	18.9	1.08
Patient 27				
Time 1	3.4	52.0	33.3	1.15
Time 2	330.2	70.1	19.8	0.85
Patient 28				
Time 1	852.4	66.3	24.7	0.74
Time 2	1299.7	85.0	8.5	0.36

## Discussion

The lungs are more vulnerable to SARS-CoV-2 than other organs. Pathological studies have shown that the primary changes in the lungs of COVID-19 patients include diffuse alveolar injury, fibrinous protein exudation, and alveolar cell desquamation accompanied by transparent membrane formation and lymphocyte-based inflammatory cell infiltration in the stroma [12]. A handful of autopsy reports have further demonstrated that COVID-19 is a disease that induces multi-organ and multi-system damage and does not simply affect the lungs [13]. The immune system damage caused by SARS-CoV-2 should not be underestimated. The spleen volume of COVID-19 patients is significantly reduced, and the number of CD4^+^ T cells and CD8^+^ T cells in the spleen and peripheral lymph nodes is also reduced; such effects are accompanied by tissue degeneration and necrosis as well as a proliferation of macrophages, which are specifically like those noted with SARS-CoV infection [14–16]. Lymphocytopenia seems to potentially signify that COVID-19 may deplete and disrupt the immune system in some direct or indirect manner, resulting in an AIDS-like response. Studies on SARS indicate that SARS-CoV cannot infect human lymphocytes and monocytes in vitro and that attacking lymphocytes and mononuclear cells with infectious SARS-CoV, inactivated virus particles, or receptor protein-binding fragments of the virus is unable to trigger an apoptotic response [17]. Besides, autopsies of COVID-19 patients yielded negative immunohistochemistry and PCR results from spleen, bone marrow, and peripheral lymphoid tissues. The above results suggest that SARS-CoV-2 is unlikely to destroy the human immune system via a direct mechanism. The reason is probably related to the lack of angiotensin-converting enzyme 2 (ACE2) expression in human immune tissues or organs [18, 19].

However, the mechanism underlying lymphopenia in peripheral blood of COVID-19 patients remains unclear. There are three possible explanations: a) The inflammatory storm gives rise to the destruction and consumption of lymphocytes. Studies have shown that the strong type I interferon (IFN) response caused by a viral infection and the high levels of glucocorticoids caused by normal stress responses can induce T cell apoptosis [20, 21]. Also, the intense cytokine storm itself experienced by SARS patients can induce lymphocyte apoptosis [17, 22], suggesting that lymphocyte apoptosis might exist in COVID-19 patients. b) Reduced lymphocyte production. Any debilitating disease inevitably activates the stress response mediated by the hypothalamic-pituitary-adrenal (HPA) axis and increases cortisol secretion. Steroid levels in the blood can significantly affect the number and biological behaviour of lymphocytes in the circulatory system. Robertson et al. reported that glucocorticoids can induce human lymphoblast apoptosis; even under physiological conditions, the number of lymphocytes also has a significant negative correlation with the circadian rhythm of the cortisol content [23–25]. After viral infection, the body exhibits a stress reaction, and the HPA axis is activated to produce more steroids, thus inhibiting the level of lymphocytes in the circulatory system. Of course, the possibility that the lymphocyte levels also change after patients receive an exogenous cortisol treatment cannot be excluded. However, many COVID-19 patients have exhibited a decreasing trend in lymphocytes in peripheral blood before receiving a clinical intervention. At present, controversy exists regarding whether glucocorticoids should be used to relieve the symptoms of patients with severe viral pneumonia [7, 26–28]. The application of glucocorticoids in the treatment of COVID-19 should be considered dialectically. Besides, whether glucocorticoids can cause immunosuppression in SARS-CoV-2-infected patients and the relationship between the dosage of glucocorticoids and the prognosis of patients still require further research. c) Abnormal distribution of lymphocytes in the body. The immune response of the respiratory tract to invasive pathogens is initiated by airway epithelial cells. After airway epithelial cell infection, resident respiratory dendritic cells (DCs) are activated by pathogens or antigens to process antigens and simultaneously migrate to peripheral lymphoid organs. After arriving at peripheral lymphoid organs, DCs present the processed antigens to immature T lymphocytes in the form of the major histocompatibility complex (MHC)-peptide complex. After binding to the MHC-peptide complex, T cells are activated to proliferate and migrate to the infected site [29, 30]. This process will inevitably lead to the redistribution of lymphocytes in the lesion and other areas. It is worth noting that the effect of COVID-19 on the immune system does not simply involve reducing the number of lymphocytes via a specific mechanism, which is quite likely attributed to a combination of the above three reasons. The exact mechanism of this change needs to be confirmed by relevant cellular and molecular pathology research. In addition, our study also demonstrated that the number and percentage of lymphocytes decreased progressively as COVID-19 progressed, suggesting that the level of lymphocytes in the blood might be a biomarker to predict the prognosis of COVID-19 patients.

Our study also found a significant positive correlation between the percentage of neutrophils in peripheral blood and the severity of pulmonary infection. Neutrophils are differentiated from hematopoietic stem cells in the bone marrow and exhibit active chemotaxis, phagocytosis, and bactericidal effects. As the most abundant leukocytes in the circulatory system, neutrophils play a central role in the natural immune system and participate in the regulation of adaptive immune responses. Generally, neutrophil activation is more sensitive to bacterial infection, but research on SARS has shown that cytokines and complement activation play an important role in the progression of SARS, which is related to neutrophil activation and aggregation [31, 32]. Based on this finding, it is hypothesised that the increased proportion of neutrophils in peripheral blood of patients with COVID-19 may also be related to the production of multiple cytokines (such as IFN-γ) and the activation of the complement system after infection with the virus. Moreover, if the patient is infected with bacteria in the late stage of the disease, the percentage of neutrophils would also increase. A retrospective study involving 1312 patients with SARS reported that the neutrophil count is a highly reliable prognostic indicator of fatality in SARS-CoV-infected patients, predicting relatively high mortality [33]. Evidence also suggests that when people are infected with some severe respiratory viruses (such as SARS-CoV, H5N1), neutrophil infiltration into the lungs will produce high levels of chemokines, such as C-X-C motif chemokine 10 (CXCL10), which can induce fulminant pneumonia and aggravate the ARDS [34]. Based on the above reasons, we suggest that the neutrophil level in peripheral blood should be an area of focus during the treatment of COVID-19 patients. Once the neutrophil level in peripheral blood becomes abnormal, certain interventions and related supportive treatment should be administered in time to improve the prognosis and to reduce the fatality rate.

Our research has the following limitations. First, the sample size is small, and the data are not normally distributed. Thus, the information obtained may exhibit deviations. Subsequent research with a larger sample is needed to further reveal the specific relationship between these two factors. Second, the inconsistency in the treatment options of the patients included is also another limitation. Although it has been confirmed that the lymphocyte count in COVID-19 patients is reduced before they receive treatment, it cannot be excluded that different treatment options administered during hospitalization could bias the results.

## Conclusions

In this study, AI was used to assess the extent of pulmonary lesions in COVID-19 patients, and the correlation between overall lung lesions and related clinical laboratory tests was analysed, revealing that the reduction in the peripheral blood lymphocyte level and the increase in the neutrophil level caused by COVID-19 were significantly related to the degree of lung lesions. Moreover, the dynamic changes of the two indicators after SARS-CoV-2 infection could play a role in guiding the choice of treatment options for patients.

## Supplementary Information

**Additional file 1.** Overal pulmonary infection results in 31 patients with COVID-19 calculated by AI system.

**Additional file 2.** Clinical laboratory examination results of 31 patients with COVID-19 corresponding to pulmonary VOI and POI at the same stage of the disease.

## Data Availability

The datasets collected and analysed during the current study are included in this published article and its supplementary information files. More detail information on the datasets and materials used in this study are available from the corresponding author on reasonable request.

## Abbreviations

COVID-19: Coronavirus disease 2019
SARS-CoV-2: Severe acute respiratory syndrome coronavirus type 2
MERS: Middle East respiratory syndrome
ARDS: Acute respiratory distress syndrome
WHO: World Health Organization
CT: Computer tomography
AI: Artificial intelligence
DL: Deep learning
RT-PCR: Reverse-transcription polymerase chain reaction
HRCT: High-resolution CT
ncpDTP-7: New coronavirus pneumonia diagnosis and treatment plan (trial version 7)
PACS: picture archiving and communication system
GGO: Ground-glass opacity
HU: Hounsfield unit
VOI: Volume of infection
POI: Percentage of infection
WBC: White blood cell
N%: Neutrophil percentage
L%: Lymphocyte percentage
M%: Monocyte percentage
LY: Lymphocyte
HGB: Haemoglobin
ACE2: Angiotensin-converting enzyme 2
IFN: Interferon
HPA: Hypothalamic-pituitary-adrenal
DCs: Dendritic cells
MHC: Major histocompatibility complex
CXCL10: C-X-C motif chemokine 10
