# Supplementary material for: Correlation between lung infection severity and clinical laboratory indicators in patients with COVID-19: a cross-sectional study based on machine learning
Source: BMC Infect Dis. 2021 Feb 18;21:192. doi: 10.1186/s12879-021-05839-9 (PMC7891484; doi:10.1186/s12879-021-05839-9)
Supplement: Supplementary file 1 — Additional file 1. Overal pulmonary infection results in 31 patients with COVID-19 calculated by AI system. [file 12879_2021_5839_MOESM1_ESM.docx]

**Additional file 1** Overal pulmonary infection results in 31 patients with COVID-19 calculated by AI system

|  | | | | | | | VOI (cm^3^) | | | | | POI (%) | | | | |
| --- | --- | --- | --- | --- | --- | --- | --- | --- | --- | --- | --- | --- | --- | --- | --- | --- |
| Patient number | WL-  VOI | WL-POI | LL-VOI | LL-POI | RL-VOI | RL-POI | LUL | LLL | RUL | RML | RLL | LUL | LLL | RUL | RML | RLL |
| 1 | 63.7 | 1.4 | 63.7 | 3.1 | 0 | 0 | 0.7 | 63 | 0 | 0 | 0 | 0.1 | 6.8 | 0 | 0 | 0 |
| 2 | 42.7 | 1 | 1.2 | 0.1 | 41.6 | 1.8 | 0.1 | 1.1 | 0 | 0 | 41.6 | 0 | 0.1 | 0 | 0 | 3.7 |
| 3 | 29.8 | 0.5 | 0 | 0 | 29.8 | 0.9 | 0 | 0 | 0 | 0 | 29.8 | 0 | 0 | 0 | 0 | 1.8 |
| 4 | 1876.7 | 63.8 | 835.4 | 57.1 | 1041.3 | 70.5 | 510.4 | 325 | 341.9 | 181.7 | 517.7 | 52.5 | 100 | 48.1 | 84.6 | 100 |
| 5 | 800.1 | 39.2 | 329.3 | 33.3 | 470.8 | 44.7 | 248.4 | 80.9 | 196.2 | 98.4 | 176.3 | 52.4 | 18 | 72.4 | 55.8 | 44.3 |
| 6 | 264.8 | 9 | 135.7 | 10.2 | 129 | 8 | 66.2 | 69.5 | 92.8 | 2.3 | 34 | 7.9 | 14.9 | 13 | 0.7 | 6.4 |
| 7 | 9.1 | 0.2 | 2.9 | 0.1 | 6.2 | 0.2 | 0.1 | 2.8 | 0 | 0 | 6.2 | 0 | 0.2 | 0 | 0 | 0.4 |
| 8 | 159.4 | 2.6 | 123.2 | 4.5 | 36.2 | 1.1 | 62.4 | 60.8 | 4.3 | 1.7 | 30.2 | 4.9 | 4.2 | 0.4 | 0.3 | 1.9 |
| 9 | 23.5 | 0.5 | 0 | 0 | 23.5 | 0.9 | 0 | 0 | 0 | 0.8 | 22.7 | 0 | 0 | 0 | 0.2 | 1.7 |
| 10 | 144 | 2.8 | 0.6 | 0 | 143.4 | 5.2 | 0 | 0.6 | 0.2 | 16.9 | 126.4 | 0 | 0.1 | 0 | 4.4 | 11.3 |
| 11 | 59.7 | 1.7 | 15.7 | 0.9 | 44 | 2.6 | 0.2 | 15.5 | 1.7 | 0.5 | 41.8 | 0 | 1.6 | 0.2 | 0.1 | 6.2 |
| 12 | 98.8 | 1.5 | 2.9 | 0.1 | 95.9 | 2.7 | 2.9 | 0 | 62 | 0.4 | 33.4 | 0.2 | 0 | 5.2 | 0.1 | 2 |
| 13 | 3.9 | 0.1 | 3.9 | 0.2 | 0 | 0 | 0.3 | 3.6 | 0 | 0 | 0 | 0 | 0.5 | 0 | 0 | 0 |
| 14 | 45.9 | 1.6 | 45.9 | 3.6 | 0 | 0 | 0 | 45.9 | 0 | 0 | 0 | 0 | 8.2 | 0 | 0 | 0 |
| 15 | 98.6 | 3.6 | 45.7 | 3.5 | 52.8 | 3.6 | 10.3 | 35.4 | 0.1 | 8.9 | 43.8 | 1.5 | 6.1 | 0 | 5.3 | 6.1 |
| 16 | 76.1 | 2.3 | 13.8 | 0.9 | 62.3 | 3.6 | 1.6 | 12.2 | 1.1 | 0.5 | 60.7 | 0.2 | 1.7 | 0.2 | 0.1 | 8.3 |
| 17 | 72.5 | 1 | 24.5 | 0.7 | 48 | 1.2 | 20.1 | 4.4 | 42.4 | 1.6 | 4 | 1.2 | 0.3 | 3 | 0.2 | 0.2 |
| 18 | 572.2 | 17.6 | 269.6 | 17.6 | 302.6 | 17.6 | 153.6 | 116 | 147.6 | 29.9 | 125.1 | 20.5 | 15 | 29 | 10.4 | 13.4 |
| 19 | 819.1 | 16.4 | 557.2 | 27.1 | 261.9 | 8.9 | 175.9 | 381.3 | 28.9 | 28.8 | 204.2 | 14.2 | 47.7 | 2.6 | 5.6 | 15.5 |
| 20 | 149.8 | 3.9 | 27.7 | 1.5 | 122 | 6.3 | 0 | 27.7 | 0.3 | 0 | 121.8 | 0 | 3 | 0 | 0 | 16.8 |
| 21 | 944.1 | 36.9 | 286.8 | 24 | 657.3 | 48.1 | 155.5 | 131.3 | 240.6 | 54.7 | 362 | 40.6 | 17.1 | 53.1 | 20.1 | 63.3 |
| 22 | 298.8 | 6 | 54.8 | 2.2 | 244 | 10.1 | 13.9 | 40.9 | 132.3 | 18.3 | 93.5 | 0.8 | 4.9 | 13.8 | 3.4 | 10.3 |
| 23 | 815.7 | 22.2 | 342.5 | 20.7 | 473.2 | 23.4 | 136.7 | 205.8 | 126.8 | 75.1 | 271.3 | 12.8 | 36.4 | 11.3 | 27 | 47.5 |
| 24 | 198.3 | 5.8 | 102.9 | 6.8 | 95.4 | 5 | 72.6 | 30.3 | 42.2 | 32.8 | 20.4 | 8.7 | 4.6 | 5.8 | 8.8 | 2.5 |
| 25 | 0 | 0 | 0 | 0 | 0 | 0 | 0 | 0 | 0 | 0 | 0 | 0 | 0 | 0 | 0 | 0 |
| 26 | 13.1 | 0.2 | 5.1 | 0.2 | 7.9 | 0.2 | 2.6 | 2.5 | 1.6 | 0.4 | 5.9 | 0.2 | 0.2 | 0.1 | 0.1 | 0.4 |
| 27 | 3.4 | 0.1 | 1.3 | 0 | 2 | 0.1 | 1.2 | 0.1 | 1.1 | 0 | 0.9 | 0.1 | 0 | 0.1 | 0 | 0.1 |
| 28 | 852.4 | 30.9 | 269.1 | 23 | 583.2 | 36.8 | 130.2 | 138.9 | 219.5 | 77.1 | 286.6 | 20.5 | 26.8 | 35.8 | 26.7 | 42 |
| 29 | 5.6 | 0.1 | 0.4 | 0 | 5.3 | 0.2 | 0 | 0.4 | 0 | 0 | 5.3 | 0 | 0 | 0 | 0 | 0.5 |
| 30 | 165 | 3 | 17.1 | 0.6 | 147.9 | 5.5 | 1.6 | 15.5 | 2.4 | 2.9 | 142.6 | 0.1 | 1.1 | 0.3 | 0.4 | 12.3 |
| 31 | 10.2 | 0.2 | 0.1 | 0 | 10 | 0.3 | 0 | 0.1 | 0 | 0 | 10 | 0 | 0 | 0 | 0 | 0.7 |

COVID-19 coronavirus disease 2019, AI artificial intelligence, VOI volume of infection, POI percentage of infection, WL whole lung, LL left lung, RL right lung, LUL left upper lobe, LLL left lower lobe, RUL right upper lobe, RML right middle lobe, RLL right lower lobe
