# Supplementary material for: Correlation between lung infection severity and clinical laboratory indicators in patients with COVID-19: a cross-sectional study based on machine learning
Source: BMC Infect Dis. 2021 Feb 18;21:192. doi: 10.1186/s12879-021-05839-9 (PMC7891484; doi:10.1186/s12879-021-05839-9)
Supplement: Supplementary file 2 — Additional file 2. Clinical laboratory examination results of 31 patients with COVID-19 corresponding to pulmonary VOI and POI at the same stage of the disease. [file 12879_2021_5839_MOESM2_ESM.docx]

**Additional file 2** Clinical laboratory examination results of 31 patients with COVID-19 corresponding to pulmonary VOI and POI at the same stage of the disease

| Patient number | Clinical classifications according to ncpDTP-7 (mild 0, moderate 1, severe 2, critical 3) | Interval from illness onset to CT scan, days | Days of clinical intervention before CT scan | WL-VOI (cm³) | WL-POI (%) | WBC, ×10^9^/L | HGB, g/L | N% (%) | L% (%) | M% (%) | LY, ×10^9^/L | AST, U/L | ALT, U/L | CRP, mg/L | CK, U/L |
| --- | --- | --- | --- | --- | --- | --- | --- | --- | --- | --- | --- | --- | --- | --- | --- |
| 1 | 1 | 5 | 3 | 63.7 | 1.4 | 2.65 | 117 | 30.9 | 57.0 | 12.1 | 1.51 | 12.3 | 15.9 | - | 53 |
| 2 | 1 | 7 | 3 | 42.7 | 1.0 | 7.32 | 137 | 72.5 | 19.9 | 7.4 | 1.46 | 14.8 | 14.4 | - | 37 |
| 3 | 1 | 8 | 0 | 29.8 | 0.5 | 4.02 | 136 | 54.0 | 31.3 | 13.9 | 1.26 | 11.4 | 15.5 | - | 47 |
| 4 | 3 | 5 | 0 | 1876.7 | 63.8 | 4.13 | 153 | 72.2 | 16.9 | 10.2 | 0.70 | 87.0 | 91.1 | 86.1 | 634 |
| 5 | 3 | 2 | 1 | 800.1 | 39.2 | 3.62 | 105 | 80.6 | 7.2 | 12.2 | 0.26 | 16.4 | 26.3 | 97.4 | 102 |
| 6 | 2 | 10 | 0 | 264.8 | 9.0 | 3.83 | 115 | 55.0 | 26.4 | 17.8 | 1.01 | 14.3 | 28.2 | 33.2 | - |
| 7 | 1 | 12 | 5 | 9.1 | 0.2 | 4.91 | 155 | 39.4 | 46.6 | 10.6 | 2.32 | 38.7 | 22.9 | 0.8 | 76 |
| 8 | 1 | 9 | 5 | 159.4 | 2.6 | 6.42 | 166 | 52.4 | 38.8 | 7.5 | 2.48 | 42.9 | 23.4 | 8.1 | 80 |
| 9 | 1 | 3 | 0 | 23.5 | 0.5 | 4.27 | 124 | 56.1 | 34.1 | 9.3 | 1.47 | 49.2 | 27.2 | - | 249 |
| 10 | 1 | 10 | 7 | 144.0 | 2.8 | 5.51 | 144 | 53.5 | 24.1 | 18.1 | 1.33 | 20.7 | 17.0 | 1.1 | 36 |
| 11 | 1 | 9 | 3 | 59.7 | 1.7 | 3.20 | 148 | 34.4 | 47.3 | 17.7 | 1.50 | 15.2 | 14.6 | - | 66 |
| 12 | 1 | 6 | 3 | 98.8 | 1.5 | 4.64 | 142 | 52.9 | 37.0 | 9.1 | 1.71 | 21.4 | 16.8 | - | 114 |
| 13 | 1 | 2 | 0 | 3.9 | 0.1 | 3.71 | 114 | 52.8 | 33.2 | 13.4 | 1.24 | 13.5 | 17.7 | 2.7 | 34 |
| 14 | 1 | 1 | 0 | 45.9 | 1.6 | 3.63 | 129 | 50.7 | 35.5 | 11.8 | 1.29 | 13.8 | 17.2 | 1.1 | 44 |
| 15 | 1 | 1 | 0 | 98.6 | 3.6 | 7.12 | 145 | 58.0 | 30.9 | 10.0 | 2.20 | 30.0 | 17.3 | 74.0 | 141 |
| 16 | 1 | 16 | 11 | 76.1 | 2.3 | 3.81 | 127 | 48.1 | 40.0 | 9.6 | 1.50 | 71.1 | 39.0 | - | 59 |
| 17 | 1 | 7 | 1 | 72.5 | 1.0 | 3.22 | 155 | 61.0 | 25.0 | 13.0 | 0.80 | - | - | 8.3 | 180 |
| 18 | 3 | 18 | 15 | 572.2 | 17.6 | 4.60 | 121 | 52.0 | 28.8 | 16.7 | 1.33 | 50.2 | 26.5 | 12.8 | 32 |
| 19 | 2 | 7 | 1 | 819.1 | 16.4 | 5.51 | 91 | 81.9 | 5.5 | 12.6 | 0.30 | 22.8 | 49.2 | 43.3 | 1399 |
| 20 | 1 | 12 | 2 | 149.8 | 3.9 | 2.42 | 114 | 61.9 | 29.8 | 8.2 | 0.70 | 11.4 | 17.1 | 9.8 | 58 |
| 21 | 3 | 16 | 1 | 944.1 | 36.9 | 11.3 | 134 | 92.0 | 5.1 | 2.5 | 0.59 | 18.1 | 13.3 | 9.6 | - |
| 22 | 1 | 21 | 1 | 298.8 | 6.0 | 2.90 | 123 | 60.2 | 30.3 | 8.2 | 0.88 | 47.6 | 37.7 | 6.6 | 45 |
| 23 | 2 | 17 | 7 | 815.7 | 22.2 | 7.82 | 139 | 70.0 | 18.2 | 12.0 | 1.40 | 32.3 | 15.4 | 0.8 | 20 |
| 24 | 1 | 20 | 0 | 198.3 | 5.8 | 5.81 | 107 | 67.2 | 18.9 | 13.0 | 1.08 | 35.0 | 21.2 | 2.6 | 32 |
| 25 | 1 | 11 | 1 | 0.0 | 0.0 | 5.93 | 124 | 52.0 | 40.0 | 5.7 | 2.40 | 36.9 | 19.0 | 1.4 | 100 |
| 26 | 1 | 3 | 0 | 13.1 | 0.2 | 4.52 | 141 | 46.9 | 33.0 | 14.0 | 1.50 | 52.3 | 23.3 | 1.2 | 55 |
| 27 | 1 | 3 | 0 | 3.4 | 0.1 | 3.50 | 134 | 52.0 | 33.3 | 11.0 | 1.15 | 107.2 | 27.0 | 1.4 | - |
| 28 | 1 | 5 | 2 | 852.4 | 30.9 | 2.99 | 110 | 66.3 | 24.7 | 9.0 | 0.74 | 20.4 | 32.3 | - | 132 |
| 29 | 1 | 11 | 3 | 5.6 | 0.1 | 3.63 | 125 | 32.8 | 62.0 | 4.7 | 2.20 | 16.6 | 17.9 | 1.4 | 70 |
| 30 | 2 | 5 | 3 | 165.0 | 3.0 | 5.31 | 167 | 49.1 | 39.1 | 11.0 | 2.10 | 122 | 82.3 | - | 191 |
| 31 | 1 | 12 | 4 | 10.2 | 0.2 | 6.44 | 132 | 70.0 | 20.0 | 8.2 | 1.34 | 14.2 | 15.4 | - | 66 |

COVID-19 coronavirus disease 2019, VOI volume of infection, POI percentage of infection, ncpDTP-7 new coronavirus pneumonia diagnosis and treatment plan (trial version 7), CT computer tomography, WL whole lung, WBC, white blood cell, HGB hemoglobin, N% neutrophil percentage, L% lymphocyte percentage, LY lymphocyte, AST aspartate transaminase, ALT alanine transaminase, CRP C-reactive protein, CK creatine kinase
